# Trends in the Management of Testicular Torsion: A Scoping Review of Delays and Outcomes

**DOI:** 10.7759/cureus.92624

**Published:** 2025-09-18

**Authors:** Althea O George, Adeyeye Olalekan, Raymond Omiko, Lean Al Saqer, Oshoke K Ilokhor, Prudence Ikechukwu, Minwook Lee, Ahmed Nassar

**Affiliations:** 1 Surgery/Urology, Royal London Hospital, London, GBR; 2 Urology, Surgery Interest Group of Africa, Lagos, NGA; 3 Social Policy, University of Strathclyde, Glasgow, GBR; 4 Urology, Royal London Hospital, London, GBR; 5 Psychiatry, Derbyshire Healthcare NHS Foundation Trust, Derby, GBR; 6 Oncology, The Christie NHS Trust, Manchester, GBR; 7 Urology, Barts Health NHS Trust, London, GBR

**Keywords:** acute scrotum, delayed diagnosis, delays, orchidectomy, orchidopexy, spermatic cord twist, testicular salvage, testicular torsion

## Abstract

Testicular torsion is a time-sensitive urological emergency in which delays in diagnosis and treatment can lead to testicular loss. Despite advances in healthcare delivery, delayed presentation and management remain common worldwide and contribute to significant morbidity. This scoping review aimed to explore trends in presentation, diagnosis, and surgical management of testicular torsion across healthcare settings; identify and categorise the causes of delays; assess the impact of these delays on outcomes; highlight predictive factors influencing salvage; and map gaps in the literature to inform future research and interventions. The review was conducted in accordance with the Joanna Briggs Institute methodology and Preferred Reporting Items for Systematic Reviews and Meta-Analyses Extension for Scoping Reviews (PRISMA-ScR) guidelines. PubMed, Scopus, and Google Scholar were searched for studies published between 2010 and 2025. Eligible studies were original peer-reviewed research articles reporting on delays, predictive factors, or outcomes of testicular torsion. Data were charted on study characteristics, delays, predictive factors, and surgical outcomes. Ten studies were included, representing 1,910 patients with acute scrotal pain, of whom 1,529 had confirmed torsion. Delays in presentation and diagnosis were multifactorial, arising from patient-related, diagnostic, and system-level barriers. Salvage rates varied from 12% to 82%, with an overall salvage rate of 41.5% and orchidectomy rate of 58.5%. Outcomes were closely linked to timing of surgery, with salvage highest within six hours of symptom onset and declining sharply thereafter. Predictive factors included symptom duration, degree of torsion, age, and imaging findings. None of the studies reported long-term functional outcomes. Delays remain the most important determinant of outcome in testicular torsion. Findings are consistent with international guidelines emphasising urgent exploration within six hours. Improved patient education, streamlined referral pathways, and institutional preparedness are essential to reduce preventable testicular loss. Future research should prioritise long-term outcomes and the evaluation of system-level interventions.

## Introduction and background

Testicular torsion is a urological emergency characterized by twisting of the spermatic cord, which compromises testicular blood flow and may lead to irreversible ischemia if not promptly managed [1]. It is most commonly seen in neonates and adolescents, though it can occur at any age. The time-sensitive nature of this condition has been well established in the literature, with optimal testicular salvage outcomes reported when surgical detorsion is performed within six hours of symptom onset [1,2]. Beyond this "golden window," the likelihood of testicular viability declines sharply, increasing the risk of orchidectomy and long-term sequelae such as infertility and psychological distress [2].

Diagnosis is primarily clinical, with hallmark features including acute onset of severe unilateral scrotal pain, swelling, and an absent cremasteric reflex. Color Doppler ultrasonography is often employed as an adjunct, as it can demonstrate absent or reduced intratesticular blood flow [3]. However, its limitations, particularly false-negative findings in cases of incomplete or intermittent torsion, mean that it should not delay surgical exploration when torsion is suspected. International guidelines, therefore, emphasize that testicular torsion remains a clinical diagnosis and that urgent exploration is required in all cases with high suspicion.

The consequences of delayed or missed management are profound, ranging from orchidectomy and reduced fertility potential to endocrine dysfunction and psychological distress [3,4]. Understanding the causes of delay and their impact on outcomes is critical to improving patient care.

Variability in institutional management strategies, the decision to explore in equivocal cases, and findings on seasonal incidence patterns further emphasize the need for a comprehensive synthesis of current trends and challenges in torsion management [5]. As evidence continues to evolve, it is important to assess how these factors influence patient outcomes and inform future guidelines.

This scoping review aims to (i) explore the trends in presentation, diagnosis, and surgical management of testicular torsion across various healthcare settings, (ii) identify and categorize the causes of delays in management, including patient-related, diagnostic, and system-level factors, (iii) assess the impact of these delays on clinical outcomes, particularly testicular salvage and orchidectomy rates, (iv) highlight predictive factors associated with testicular outcomes, and (v) map existing gaps in the literature to inform future research directions and guide interventions aimed at improving timely diagnosis and care.

## Review

Methods

Review Design

This scoping review was conducted in accordance with the methodological framework proposed by the Joanna Briggs Institute (JBI) for scoping reviews [6]. The reporting was guided by the Preferred Reporting Items for Systematic Reviews and Meta-Analyses Extension for Scoping Reviews (PRISMA-ScR) checklist [7]. The aim of this review was to systematically map existing literature on testicular torsion, with particular emphasis on delays in presentation, diagnosis, and management, and their association with clinical outcomes.

Protocol and Registration

A protocol outlining the objectives, eligibility criteria, and methodology for this scoping review was developed prior to the commencement of the study. The protocol was informed by the Joanna Briggs Institute (JBI) methodology for scoping reviews and the PRISMA-ScR checklist [6,7]. Although the protocol was not registered in a public repository, all steps of the review process were guided by the JBI framework and PRISMA-ScR guidelines to ensure methodological reproducibility and transparency.

Eligibility Criteria

The inclusion and exclusion criteria were defined using the PCC (Population, Concept, Context) framework. The population considered in this review included males of any age who presented with testicular torsion. The concept focused on examining trends in diagnosis and management, identifying factors that contribute to delays, and assessing predictive factors for testicular salvage or orchidectomy, along with related clinical outcomes. The context encompassed all healthcare settings worldwide, covering both pediatric and adult populations.

This review included original peer-reviewed research articles such as retrospective and prospective cohort studies, case series, and case-control studies. Only studies published in English were considered eligible. Articles were required to address at least one of the following domains: delays in presentation, timing of diagnosis, testicular salvage outcomes, or predictive factors associated with testicular viability.

Studies were excluded if they were case reports, editorials, letters to the editor, conference abstracts, or narrative reviews. Research focusing exclusively on neonatal torsion or non-testicular torsion conditions, such as torsion of the appendix testis, was also excluded.

Information Sources and Search Strategy

A systematic literature search was conducted in PubMed, Scopus, and Google Scholar. The search strategy included MeSH terms and free-text terms such as "testicular torsion," "spermatic cord twist," "delayed diagnosis," "testicular salvage," "orchidectomy," "orchidopexy," "presentation delay," "management trends," and "outcomes." The search was restricted to articles published between January 2011 and July 2025 to capture contemporary practices and outcomes. A full search strategy is included in Appendix A.

Study Selection

**Figure 1:**
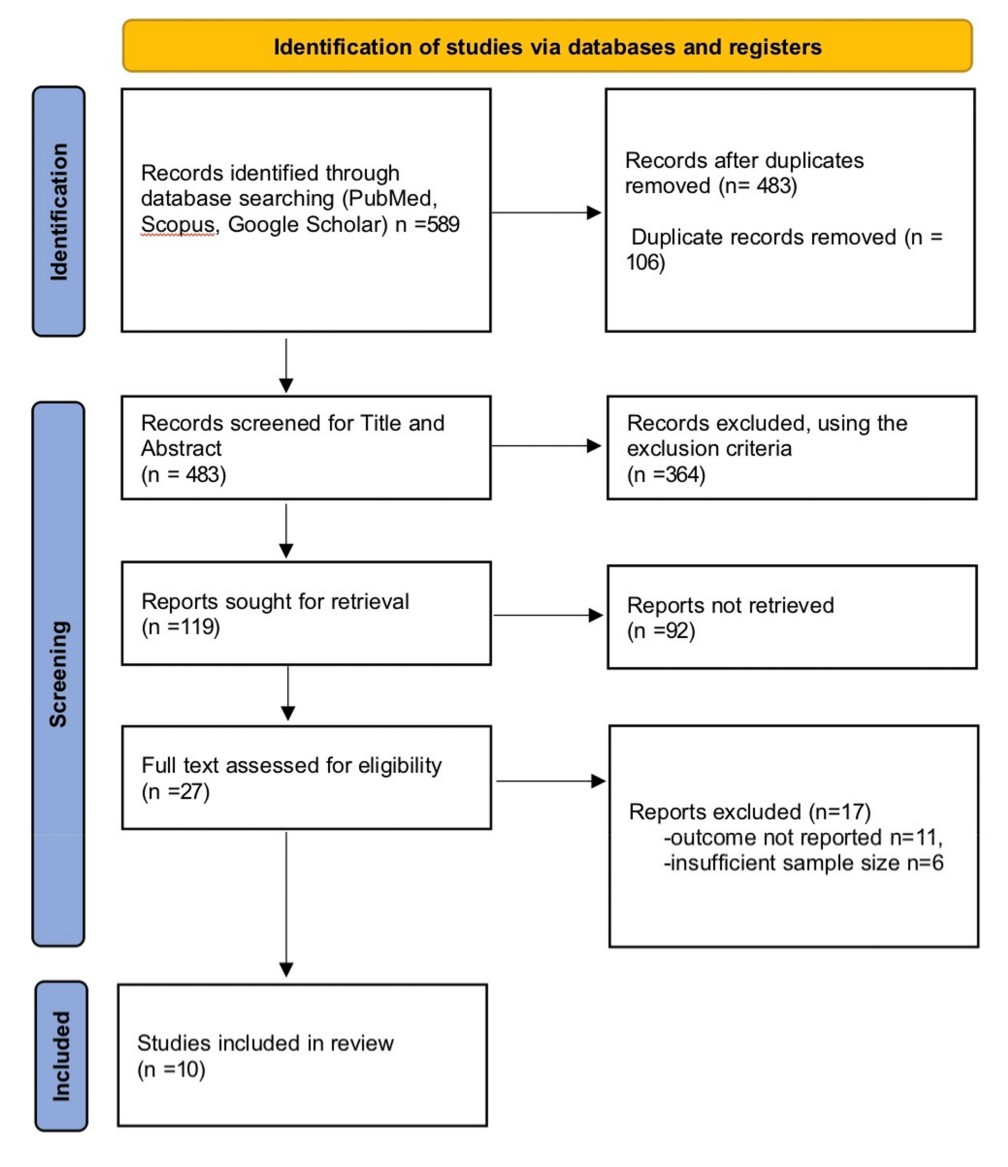
PRISMA Flow Diagram PRISMA: Preferred Reporting Items for Systematic Reviews and Meta-Analyses.

All identified records were imported into a reference manager, and duplicates were removed. Two independent reviewers screened titles and abstracts for relevance. Full texts of potentially eligible studies were reviewed against the inclusion criteria. Discrepancies were resolved through consensus. A PRISMA flow diagram (Figure 1) outlines the study selection process.

Data Charting Process

A standardized data charting form was developed to systematically extract relevant information from each included study. Key variables collected included author, year of publication, country of origin, study design, sample size, population characteristics, reported delays (for example, in presentation, diagnosis, or surgery), predictive factors (such as symptom duration or degree of torsion), and clinical outcomes like testicular salvage or orchidectomy.

Two reviewers independently charted the data, and discrepancies were resolved through discussion. Where specific data were missing or unclear, this was noted in the table. The charted information was then organized thematically to facilitate synthesis under the core domains of this review: delays in management, predictors of outcome, and trends in testicular torsion treatment. Details are provided in Appendix B.

Study Characteristics

Ten studies published between 2011 and 2025 were included, representing a total of 1,910 patients with acute scrotal pain, of whom 1,529 had confirmed testicular torsion. Most were retrospective studies conducted in tertiary or academic centers across various regions, including Europe, Asia, Africa, and South America. Two studies focused on pediatric populations, while the remainder included adolescents and young adults. Commonly reported variables were time to presentation, degree of torsion, diagnostic delays, and surgical outcomes (orchidectomy versus salvage).

Table 1 summarizes key characteristics, including author, year of publication, study location, sample size, and study design. 

**Table 1:** Study Characteristics

Author	Year of Publication	Country of Origin	Sample Size	Study Design
Tian et al. [8]	2025	China	176	Retrospective study
O’Connell et al. [9]	2025	Ireland	203	Retrospective study
Raffee et al. [10]	2024	Jordan	308	Retrospective study
Molokwu et al. [11]	2011	UK	173	Case series
Gang et al. [12]	2024	China	75	Retrospective study
Dias et al. [13]	2020	Brazil	505	Case series
Howe et al. [14]	2017	USA	81	Observational study
Ramachandra et al. [15]	2015	USA	114	Retrospective study
Murali et al. [16]	2022	India	101	Retrospective study
Kabore et al. [17]	2021	Burkina Faso	74	Case-control study

Results

Overview of Included Studies

Ten studies were included in this review, encompassing a combined total of 1,910 patients with testicular torsion. The majority of studies were retrospective in design, with one case-control study and one prospective observational study. Sample sizes ranged from 74 to 505 patients, and the populations studied included both pediatric and adult males, though four studies focused specifically on pediatric or adolescent cohorts. Most studies were conducted in tertiary referral centers across diverse geographic regions, including China, Ireland, Jordan, the United Kingdom, the United States, Brazil, India, and Burkina Faso.

Delays in Presentation and Diagnosis

Delays in presentation and diagnosis were a consistent determinant of outcomes across the included studies. Patient-related delays were frequently reported, with median symptom durations ranging from as little as four minutes in pediatric cohorts to as long as 72 hours in broader population samples. Tian et al. and Ramachandra et al. highlighted the strong association between prolonged duration of symptoms and higher rates of orchidectomy, particularly beyond 24 to 48 hours [8,15]. O’Connell et al. reported the longest delays, with a median presentation time of 72 hours, leading to orchidectomy in nearly 90% of patients [9]. This analysis identifies and categorizes patient-related, diagnostic, and system-level causes of delay, fulfilling the second objective.

System-level delays were also evident, particularly in resource-limited settings. Dias et al. described a median delay of 8.7 hours attributable to inter-hospital transfers, while misdiagnosis at the referring institution further contributed to delayed surgical intervention [13]. These findings underscore the compounding effects of both patient-level and institutional factors in determining outcomes.

Predictive Factors Influencing Testicular Salvage

Across studies, the duration of symptoms consistently emerged as the strongest predictor of testicular salvage. Patients who underwent surgery within six hours of symptom onset had the highest likelihood of preservation, while orchidectomy rates rose dramatically beyond 24 to 48 hours. The degree of torsion was also predictive, as shown by Howe et al., where twists greater than 360° correlated with non-viability [14].

Ultrasound findings emerged as complementary prognostic indicators. Tian et al. reported that of 176 patients, non-homogeneous echotexture and absent testicular blood flow on Doppler ultrasound were strongly associated with orchidectomy [8]. Specifically, 121 testes (69%) were lost, the majority of which had abnormal imaging findings, while salvage was achieved in 55 cases (31%), typically when vascularity was preserved. This illustrates that ultrasound should support, but not replace, clinical judgment in suspected torsion [8]. Age appeared variably predictive: Ramachandra et al. found higher salvage rates among patients older than eight years, while Murali et al. reported higher orchidectomy rates in patients under 18 years [15].

Diagnostic Challenges and Negative Explorations

Several studies highlighted the diagnostic challenges in distinguishing torsion from other causes of acute scrotum. Molokwu et al. reported that only 18% of patients undergoing scrotal exploration for suspected torsion required orchidectomy, with the majority presenting late [11]. Murali et al. similarly reported that of 101 patients undergoing scrotal exploration, only 64 were confirmed as torsion cases, reflecting a notable proportion of negative explorations [16].

Ultrasound added to this diagnostic complexity. Although absent blood flow and heterogeneous echotexture were predictive of orchidectomy, cases with normal or equivocal ultrasound findings were still found to have torsion at exploration. Such false negatives can delay surgical intervention, emphasizing the risk of relying solely on imaging. This reinforces the principle that ultrasound should remain an adjunct to, rather than a substitute for, clinical judgment.

Testicular Salvage and Orchidectomy Rates

Rates of testicular salvage varied substantially across the included studies, ranging from 12% in O’Connell et al. to 68% in Howe et al. [9,14]. This variation largely reflected differences in presentation time and health system efficiency. In Raffee et al.’s study, patients undergoing orchidopexy had a median presentation delay of only 13 hours, compared to 144 hours in those who required orchidectomy [10]. In the large Brazilian cohort reported by Dias et al., orchidectomy occurred in 54% of patients, while salvage was possible in 46% [13]. Similarly, Kabore et al. documented a salvage rate of 43% and orchidectomy in 57% of cases, with direct hospital admission strongly associated with better outcomes compared to referral cases [17]. These data demonstrate the impact of delays on salvage and orchidectomy rates, corresponding to the third objective. Table 2 provides a summary of the salvage versus orchidectomy rates.

**Table 2:** Salvage vs. Orchidectomy Outcomes

Study	Total Torsion Cases (n)	Testicular Salvage (n)	Orchidectomy (n)	Salvage Rate (%)	Orchidectomy Rate (%)
Tian et al. [8]	176	55	121	31	69
O’Connell et al. [9]	203	23	180	12	88
Raffee et al. [10]	308	114	194	37	63
Molokwu et al. [11]	89	73	16	82	18
Gang et al. [12]	75	43	32	57	43
Dias et al. [13]	345	159	186	46	54
Howe et al. [14]	81	55	26	68	32
Ramachandra et al. [15]	114	63	51	55.3	44.7
Murali et al. [16]	64	18	46	28.1	71.9
Kabore et al. [17]	74	32	42	43	57

Seasonal and Institutional Trends

Seasonal variation in torsion incidence was documented in multiple studies. Raffee et al. reported that torsion was most common during winter (30% of cases), while Molokwu et al. and Gang et al. similarly noted higher frequencies during colder months [10-12]. Institutional factors were also critical, particularly in studies from resource-limited settings. Dias et al. and Kabore et al. both found that inter-hospital transfer and referral patterns were strongly associated with increased delays and higher orchidectomy rates [13,17]. In contrast, patients presenting directly to tertiary centers generally had higher salvage rates. These findings reflect broader contextual and system-level factors, complementing the first and second objectives.

Discussion

This scoping review synthesized findings from 10 studies addressing the presentation, diagnosis, and management of testicular torsion, with a focus on delays and their impact on outcomes. Across the included literature, a wide variation was observed in presentation times, with median symptom duration ranging from less than one hour in some pediatric cohorts to more than 72 hours in others. Surgical exploration was consistently reported as the definitive management strategy, although diagnostic uncertainty contributed to a notable proportion of negative explorations. These findings reaffirm the principle articulated by the European Association of Urology (EAU) that surgical exploration should not be delayed in cases of high clinical suspicion, even if imaging results are equivocal [18].

Delays in management emerged as a critical determinant of outcome, arising from both patient-related and system-level factors. Late presentation was common, particularly in adolescents and adults, often due to lack of symptom awareness or reluctance to seek urgent care. Diagnostic delays were described in studies such as that of O’Connell et al., where misinterpretation of clinical findings contributed to significantly prolonged intervention times [9]. In resource-limited contexts, delays were compounded by referral pathways and inter-hospital transfers, as demonstrated by Dias et al. and Kabore et al., where indirect presentation was strongly associated with orchidectomy [13,17]. These findings highlight the multilayered contributors to poor outcomes and underscore the importance of both community education and institutional preparedness.

The impact of these delays was evident in the wide variation in reported salvage and orchidectomy rates. In studies where the median time to intervention was under 12 hours, salvage rates were generally higher than 50%, with Molokwu et al. reporting 82% [11]. Conversely, when delays extended to 24 hours or beyond, orchidectomy predominated, as in O’Connell et al., where 88% of patients lost the affected testis. These findings align with the EAU’s recognized salvage timeline: approximately 90% within the first six hours, 50% by 12 hours, and less than 10% beyond 24 hours. Such evidence reinforces the time-critical nature of torsion and the need for streamlined care pathways to facilitate rapid diagnosis and surgery [18].

These findings are consistent with established international guidelines. The EAU recommends immediate surgical exploration for all suspected cases, with salvage most likely when intervention occurs within six hours of symptom onset [18]. The AUA (American Urological Association) guidelines similarly emphasize that torsion is a surgical emergency and that imaging should not delay exploration in patients with a suggestive clinical picture [19]. The BAUS (British Association of Urological Surgeons) guidelines echo this position, reinforcing the principle of "time is testis" and advocating for urgent exploration as the standard of care [20]. Together, these guidelines provide a unified benchmark that highlights the narrow therapeutic window for intervention and the importance of minimizing both diagnostic and referral delays.

Ultrasound findings also emerged as important predictors across the literature. Absent intratesticular blood flow and heterogeneous echotexture correlated with non-viability, as reported in Tian et al., and were more frequently observed in patients undergoing orchidectomy. However, false-negative or misleading scans were described, where testes with apparent vascularity on Doppler were later confirmed to be non-viable at exploration [8]. These cases illustrate how some testes were lost due to reliance on reassuring but inaccurate imaging, further supporting the guideline recommendation that high clinical suspicion should mandate exploration irrespective of imaging findings.

When compared to international benchmarks, the findings of this review highlight clear disparities. In high-resource settings, orchidectomy rates are typically reported between 30% and 50%, whereas in many of the included studies, rates exceeded 60%, particularly in low- and middle-income countries where system-level delays, referral pathways, and limited emergency access were major contributors [8-10,16]. This variation underscores the role of health system infrastructure in determining outcomes and highlights the need for global standards that ensure equitable and timely access to emergency surgical care [21].

Several clinical predictors of testicular outcome were consistently reported across studies. Symptom duration remained the most robust predictor, while degree of torsion was also strongly associated with viability. Imaging findings such as absent blood flow or heterogeneous echotexture were useful adjuncts, though their role remains supportive rather than definitive [8,9,14]. Age demonstrated variable associations, with some cohorts suggesting higher salvage rates in older children and others reporting worse outcomes in those under 18 [16]. Collectively, these findings suggest that while clinical and imaging features may help stratify risk, timely intervention remains the most decisive factor for outcome.

Taken together, the evidence underscores the continuing importance of public health awareness, early presentation, and the adoption of efficient institutional protocols to reduce preventable testicular loss [22]. By embedding these lessons into clinical practice, healthcare systems can move closer to meeting the benchmark established by the EAU: urgent surgical exploration within six hours of symptom onset as the standard of care for all suspected cases of torsion [18].

Research Gaps and Future Directions

This review highlights several important gaps in the literature on testicular torsion. Most existing studies focus on immediate surgical outcomes, with little attention given to long-term sequelae such as fertility potential, hormonal status, and psychological impact, particularly in adolescents and young adults. The absence of such data limits understanding of the broader consequences of torsion beyond testicular viability.

A further gap lies in the inconsistent reporting of time intervals from symptom onset to presentation, diagnosis, and surgery. Standardized definitions and uniform reporting frameworks are required to enable meaningful comparison across studies and the development of evidence-based benchmarks for care. Additionally, few studies have systematically evaluated institutional or system-level contributors to delay, such as inter-hospital transfer processes, availability of on-call surgical teams, or use of structured referral pathways.

Future research should prioritize prospective, multicenter studies that incorporate long-term follow-up, adopt standardized outcome reporting, and evaluate the impact of system-level interventions on reducing delays. Such work is essential to inform guideline development and to ensure equitable, timely, and effective management of this urological emergency across diverse healthcare settings.

Strengths and Limitations of This Study

A key strength of this scoping review is its comprehensive approach to mapping the current literature on testicular torsion, with a specific focus on delays and clinical outcomes. The inclusion of diverse study populations across different healthcare settings enhances the generalizability of findings. The use of a structured search strategy, adherence to the PRISMA-ScR guidelines, and a transparent data charting process further contribute to the methodological rigor of the review.

However, this study is not without limitations. The included studies were largely retrospective in design, with variable definitions of delay and inconsistent reporting of outcome measures, which may affect the robustness of cross-study comparisons. Additionally, publication bias may be present, as only English-language articles were included. Finally, while efforts were made to capture a wide range of relevant literature, some studies may have been missed due to limited access to gray literature or unpublished institutional data.

## Conclusions

This scoping review highlights that delays in the presentation, diagnosis, and surgical management of testicular torsion remain the most important determinants of outcome. Testicular salvage is highest when intervention occurs within the first six hours and declines steeply with prolonged ischemia, reflecting the narrow therapeutic window emphasized by international guidelines. Patient-related, diagnostic, and system-level factors all contribute to these delays, and predictive variables such as symptom duration and degree of torsion remain consistent across studies. While the immediate surgical outcomes are well documented, significant gaps remain regarding long-term functional consequences and the impact of institutional protocols. Improving awareness, streamlining referral pathways, and prioritizing rapid surgical exploration are essential to reducing preventable testicular loss and optimizing care for this urological emergency.

## Appendices

Appendix A 

**Table 3:** Search Strategy

Database	Search Terms/Query	Filters Applied	Date of Search
PubMed (MEDLINE)	("testicular torsion"[MeSH Terms] OR "testicular torsion"[All Fields]) AND ("delayed diagnosis" OR "presentation delay" OR "time to intervention" OR "orchidectomy" OR "testicular salvage")	English, Human, 2011–2025, Full Texts	2 August 2025
Scopus	TITLE-ABS-KEY ("testicular torsion") AND ("delayed diagnosis" OR "presentation delay" OR "time to surgery" OR "orchidectomy" OR "testicular salvage" OR "predictive factors")	English, 2011–2025, Full Texts	3 August 2025
Google Scholar	"testicular torsion" AND ("delayed diagnosis" OR "testicular salvage" OR "orchidectomy")	English, 2011–2025, Full Texts	5 August 2025

Appendix B 

**Table 4:** Data Charting Process

Author(s) and Year	Country of Study	Study Design	Population	Sample Size	Average Time	Delay Factors	Predictive Factors	Outcome
Tian et al. (2025) [8]	China	Retrospective study	Paediatric patients	176	The average symptom duration was 14 hours	Clinical and imaging-related	Imaging findings, symptom duration	31% of patients had testicular salvage, and 69% had orchidectomy. There was an increased orchidectomy rate over time, with 35.7% within 0-6 hours, 52.2% within 6-48 hours, and 95.8% beyond 48 hours.
O’Connell et al. (2025) [9]	Ireland	Retrospective case series	All age groups	203	Median time of presentation from the onset of pain was 72 hours	Presentation and diagnostic delay	Imaging findings, symptom duration	88% of patients had orchidectomy, and 12% had salvageable testes
Raffee et al. (2024) [10]	Jordan	Retrospective study	All age groups	308	Median time for orchidopexy was 13 hours. Median time for orchidectomy was 144 hours	Not specified	Season and symptom duration	30% of testicular torsions occurred during the winter period, 25% in Autumn, 24% in Spring, and 19% in summer. 37% of participants had testicular salvage with an average time of 13 hours, while 63% had orchidectomy with an average time of 14 hours
Molokwu et al. (2011) [11]	UK	Case series	All age groups	173	Median duration from onset of symptoms to presentation was 48 hours	Not specified	Seasonal variation and symptom duration	18% of patients with testicular torsion had orchidectomy, with the majority presenting >24 hours from the onset of pain. There was a higher frequency of testicular torsion in colder months compared to warmer months
Gang et al. (2024) [12]	China	Retrospective study	Infancy to adolescence	75	The average time from the event to hospitalisation ranged from 4 to 115 minutes	Time to presentation	Duration of symptoms and Seasonal variation	34 cases of testicular torsion in winter, 18 in spring, 12 in autumn, and 11 in summer. Orchidectomy rate was 43%, while salvageable testes rate was 57%. 50% of patients who had orchidectomy visited a primary hospital initially before going to a tertiary hospital, and 80% of those with salvageable testis came straight to the tertiary hospital
Dias et al. (2020) [13]	Brazil	Case series	All age groups	505	Not specified	Misdiagnosis, inter-hospital transfer	Transfer time, misdiagnosis	Median presentation delay of 8.7 hours due to inter-hospital transfer. Salvageable rate was 46%, orchidectomy was 54%. 31.7% of patients were initially misdiagnosed, leading to an increased rate of orchidectomy
Howe et al. (2017) [14]	USA	Observational study	Children and adolescents	81	Median time from onset of symptoms to surgery was 6 (1-24) hours	Symptom duration	Symptom duration, degree of torsion	68% of patients had salvageable testes, 32% had orchidectomy
Ramachandra et al. (2015) [15]	USA	Retrospective study	Paediatric patients	114	Median duration of symptoms before ED presentation was 7 hours, and an extra 3.33 hours till intervention	Presentation delay	Timing, age	55.3% of patients had salvageable testes with a median time of 4 hours, while 44.7% of patients had orchidectomy with a median time of 42 hours. Patients older than 8 years of age were more likely to undergo orchidopexy than those younger than 8 (61.5% vs 30.4%)
Murali et al. (2022) [16]	India	Retrospective study	All age groups	101	Median symptom duration was 36 hours	Not specified	Symptom duration and age	64 patients of the 101 patients had Testicular Torsion, 72% of them underwent orchidectomy with a median symptom duration of 72 hours, while 18% had salvageable testes with a median time of 6.5 hours. Orchidectomy rates were higher in those below 18 years (75%) compared to those above 18 years (66.7%)
Kabore et al. (2021) [17]	Burkina Faso	Case-control study	All age groups	74	The median duration of symptoms was 24 hours	Time to presentation	Symptom duration, Time till Intervention	Salvageable rate was 43.2% and orchidectomy rate 56.8%. Direct hospital admission had a lower orchidectomy rate (38.9%) compared to being referred, with a rate of 62.5%
